# Improving Access and Recruitment to Clinical Trials for Lung Cancer Patients: A Multi‐Phase, Qualitative Focus Group and Co‐Production Study

**DOI:** 10.1111/jan.70134

**Published:** 2025-08-14

**Authors:** Christopher Dodd, Benjamin Lond, Zoe Davey, Iain R. Williamson, Liz Darlison, Sally Hall, John McPhelim, Janette Rawlinson, Catherine Henshall

**Affiliations:** ^1^ Oxford Institute of Applied Health Research, Faculty of Health, Science and Technology Oxford Brookes University Oxford UK; ^2^ De Montfort University Leicester UK; ^3^ University Hospitals of Leicester NHS Trust Leicester UK; ^4^ BTOG Research Group Loughborough UK; ^5^ University Hospital Hairmyres East Kilbride UK; ^6^ European Lung Foundation Sheffield UK; ^7^ Oxford Health NHS Foundation Trust The Warneford Hospital Oxford UK

**Keywords:** cancer, nurse roles, nurse—Patient interaction, nurse—Patient relationships, respiratory nursing

## Abstract

**Aim:**

To design and develop a novel co‐produced intervention tool aimed at facilitating discussions that lung cancer nurses have with lung cancer patients about clinical trial opportunities; and promote trial recruitment.

**Design:**

A multi‐phase qualitative focus group (phase 1) and co‐production (phase 2) study.

**Methods:**

The rigorous design and content of the intervention tool was informed by qualitative data from seven focus groups with lung cancer healthcare professionals (*n* = 38) and patients and their carers (*n* = 22) to establish barriers and facilitators to clinical trial participation. Data collection took place across England and Scotland between October and December 2023. Findings from a previously published systematic review were also incorporated to inform intervention tool design. The tool was developed through an extended co‐production workshop comprising lung cancer nurses (*n* = 7), lung cancer patients (*n* = 2) and health researchers (*n* = 4). The COM‐B model of behavioural change underpinned both phases of the project to guide tool development.

**Results:**

Phase 1 focus groups identified the need for a tool to provide basic trial information to patients, and to support lung cancer nurses in discussing trials with patients, thus improving nurses' knowledge, confidence, and awareness of trials. The phase 2 coproduction workshop identified that the tool should consist of two elements: a patient‐facing information pamphlet and a large poster for nurses to assist them in discussing trial opportunities.

**Conclusion:**

The study results demonstrate how nurses can be supported to discuss clinical trial opportunities with patients, with the potential to increase long‐term recruitment to clinical trials.

**Implications for the Profession and/or Patient Care:**

Lung cancer nurses often lack confidence to support patients to make informed choices about trial enrolment. By addressing this issue, participation in lung cancer clinical trials can be significantly improved to benefit patient outcomes and trial participation rates.

**Impact:**

The tool has the potential to be used across a range of different cancer settings and sites to increase recruitment to clinical trials.

**Reporting Method:**

The COREQ checklist was utilised to ensure that robust processes were followed and reported on.

**Patient and Public Involvement:**

Patients and members of the public were involved in all study processes and contributed to the study design, interpretation of the data, and intervention design. Their contributions included reviewing focus group topic guides, reviewing data analysis, the co‐production of the intervention tool, and co‐authoring this paper, ensuring the research addressed the needs and priorities of lung cancer patients when making an informed choice about clinical trial participation.

**Summary**

- Deeper understanding of the barriers to nurse‐led conversations with patients and carers about clinical trial enrolment.
- A rigorous methodological approach to tool development, underpinned by behavioural change theory, that has replicability potential.
- The resulting tool can be used by nurses to increase recruitment to clinical trials; it has potential to be universal in its application across clinical settings.

## 1 Introduction

Lung cancer clinical trials can have a positive impact on survival rates and quality of life for people living with lung cancer (World Health Organisation 2023). Increasing the number of people enrolling on clinical trials has been identified as a priority for governments around the world, including the United Kingdom (UK) (Health and Social Care Committee 2022). The recently published O'Shaughnessy review cited a clear need to increase the number of conversations about clinical trial participation taking place between patients and the healthcare workforce, to drive forward growth in the UK Life Sciences Industry (Office for Life Sciences 2023). Despite this, patients often report that they have not had a conversation with a healthcare professional about research participation. Lung cancer nurses can address this issue, and this paper describes the development of the Lung I‐ACT (Improving Access to Clinical Trials) tool. This tool has two elements, a patient‐facing leaflet and a nurse‐facing poster, and has been developed to aid facilitation of discussions lung cancer nurses have with lung cancer patients about clinical trials opportunities. The intervention tool design was underpinned by findings from a systematic review (Lond et al. 2024) and qualitative focus groups, which provided insights into some of the barriers and facilitators to clinical trial entry. These data were combined, alongside extensive feedback from key stakeholders, to inform tool development. This iterative process enabled continuous redesign and refinement of the Lung I‐ACT tool, in line with Medical Research Council guidance on developing complex interventions (Skivington et al. 2021; O'Cathain et al. 2019).

## 2 Background

Globally, lung cancer is the second most diagnosed cancer (11.4%) and has the highest mortality rate of all cancers (18.0%) (Sung et al. 2021). In the UK, lung cancer is the third most common cancer type, affecting around 48,500 new patients each year (Cancer Research UK 2025).

Clinical trials can boost the health economies of nations through government partnerships with the life sciences industry and academic institutions; they also allow patients access to novel treatments and contribute to advances in treatment outcomes (Health and Social Care Committee 2022). As well as offering hope for patients undergoing lung cancer treatment, clinical trials are often undertaken for altruistic reasons, with the knowledge that future generations may benefit from participation being a common incentive for people to participate (Lond et al. 2024). The UK Government has stated its commitment to establishing the UK as a life sciences superpower; this relies on increased participation in clinical trials.

Despite this enthusiasm, enrolment to cancer clinical trials remains low globally. Attempts to quantify the number of cancer patients enrolled in clinical trials vary, but consistently report a figure below 10%, sometimes as low as 2%–3% (Unger et al. 2019). In the UK, a survey of 500 cancer patients found that 11% had taken part in a clinical trial, despite 95% expressing an interest in participation (Institute of Cancer Research 2021). Within lung cancer specifically, there is limited research to elucidate the proportion of patients participating in trials, but records suggest it is below 10% (Spiro et al. 2000). We do know that 12,792 participants (including patients, carers and healthcare professionals) were recruited to lung cancer research in 2023/24. This compares unfavourably with breast cancer which, despite having similar incidence levels, recruited 34,322 participants in the same year (NIHR 2024). Furthermore, only 53% of lung cancer research studies recruited to target and time in 2023/24 (NIHR 2024). The most recent data suggests there are approximately 61,000 people living with lung cancer in England (National Disease Registration Service 2021) and it is imperative that these people are aware of clinical trial opportunities that have the potential to make a difference to their quality of care and survival outcomes (Dodd et al. 2024).

Research has shown that discussions with healthcare professionals can substantially inform patient decision‐making regarding clinical trial entry (Nipp et al. 2019; Institute of Cancer Research 2021; Office for Life Sciences 2023; Lond et al. 2024). Despite this, year on year, only around half of cancer patients report having had a conversation about participation in research (NHS England 2023). A failure to discuss clinical trials with patients can lead to misconceptions about the nature of participation and create unnecessary barriers to enrolment (Nipp et al. 2019; Lond et al. 2024). Lung cancer nurses have the potential to break down these barriers due to the often close and trusting relationships that they develop with lung cancer patients and their families or carers over time (Collet et al. 2022; Dodd et al. 2024; Lond et al. 2024). Lung cancer nurses have specialist skills in caring for lung cancer patients and contribute to decisions about assessment and care planning, help to coordinate patient pathways, and are a named point of contact for lung cancer patients. However, due to shortages within the nursing workforce, lung cancer nurses are often left short of time and resources (Alessy et al. 2022). This can reduce their ability and drive to initiate conversations about clinical trial entry (McPhelim 2015). Lung cancer nurses have reported that they do not routinely discuss clinical trial entry with lung cancer patients, often citing lack of confidence, knowledge, experience, and time as contributing factors (McPhelim 2015). In addition, structural barriers, including limited communication between clinical care and research teams, reduced staff numbers, and a reliance on certain healthcare professionals to provide information about clinical trials, can make cancer nurses less likely to raise the topic of clinical trial entry with patients (Lavender and Croudass 2019).

One way to facilitate discussions between lung cancer nurses and patients about clinical trial entry is through the introduction of a bespoke educational and informational tool. The UK's National Institute for Health and Care Excellence recommends that people with lung cancer should be given accurate, easy to understand information and that they should have details about any potential treatments clearly explained to them (NICE 2024). Educational and informational tools are known to aid patient decision‐making surrounding treatment options. They can be used to provide prompts to encourage guidance around certain behaviours and contain relevant content to help guide desired actions (Michie et al. 2011). The development of a tool that can serve as an educational, decision‐making aid for patients, whilst also providing nurses with the knowledge, confidence, and awareness to initiate discussions with patients about clinical trial entry has the potential to contribute to improved rates of recruitment to lung cancer clinical trials.

## 3 The Study

To design and develop a novel co‐produced intervention tool aimed at facilitating discussions that lung cancer nurses have with lung cancer patients about clinical trial opportunities and to promote trial recruitment. The aim was underpinned by the following objectives:

- To conduct focus groups with lung cancer patients, carers, and healthcare professionals to identify barriers and facilitators to clinical trial entry.
- To synthesise focus group data with a previously published systematic review to provide evidence on barriers and facilitators to clinical trial entry and to inform tool design and development.
- To map findings against the theoretical COM‐B behaviour change model.
- To hold a co‐production workshop with UK‐wide stakeholders to inform tool development.
- Using logic modelling, work with stakeholders and study collaborators to develop a co‐produced Lung I‐ACT tool.

**FIGURE 1:**
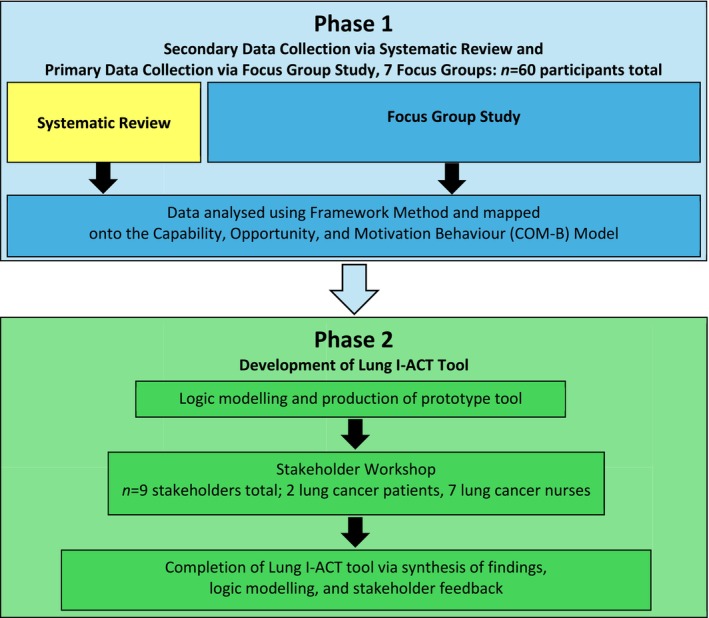
Study flow diagram showing the individual development phases of the Lung I‐ACT tool.

The phases reported on in this paper are qualitative in design and consisted of focus groups (phase 1) and a co‐production workshop (phase 2) to develop the Lung I‐ACT tool. The study activities took place between September 2023 and July 2024. The study was part of the larger Lung I‐ACT study, a UK‐based mixed‐methods, multi‐site, complex intervention study which also involved a systematic review exploring barriers and enablers to clinical trial entry for lung cancer patients (Lond et al. 2024) and the implementation and piloting of the Lung I‐ACT tool reported on in this paper (Figure 1).

## 4 Phase 1: Focus Groups

### 4.1 Methods

#### 4.1.1 Design

The study was approached through the theoretical lens of pragmatism, with an emphasis on what works best in practice. It employed a combination of both inductive and deductive approaches to data collection and analysis (Dolan et al. 2022). Online focus groups were conducted to explore lung cancer nurse, multidisciplinary team member, patient and carer perspectives regarding barriers and enablers to discussing clinical trial entry. Potential features of a tool aimed at improving the discussions nurses have with lung cancer patients about clinical trial opportunities were discussed.

#### 4.1.2 Theoretical Framework

Data were mapped onto the ‘Capability, Opportunity, and Motivation Behaviour’ (COM‐B) Model (Michie et al. 2011), to help interpret findings using an integrative framework for understanding target behaviours and intervention design. The COM‐B model is a behaviour change framework that proposes three necessary components—capability, opportunity, and motivation—for any behaviour to occur. Assessing capability, opportunity, and motivation enhances our understanding of why and how specific behaviours occur. This can enable targeted interventions to be developed to facilitate effective change (Michie et al. 2011).

#### 4.1.3 Study Setting and Recruitment

Participants were purposively recruited from eight NHS participating sites across England (*n* = 7) and Scotland (*n* = 1). This included a mixture of teaching hospitals and District General Hospitals. The seven sites in England were geographically varied and spread across the country to ensure, as far as possible, that patients and healthcare professionals with experiences of different healthcare systems and structures, as well as different socio‐demographic backgrounds, were included. Two of the English NHS sites were cancer centres of excellence and more experienced in recruiting lung cancer patients to clinical trials than the other six sites. For this reason, separate focus groups were held for healthcare professional participants from these two centres so that any data generated could be distinguished from the other sites and to ensure that the recruitment experiences articulated did not deter the views and perspectives from the other centres from being voiced. Separate online focus groups were also held for healthcare professionals and for lung cancer patients and their carers to remove any perceived hierarchical and/or professional/personal barriers that might inhibit participants from speaking freely.

Localised versions of invitation letters and participant information sheets (for healthcare professionals, patients, and carers) and a consent form were provided to the local Principal Investigator at each site who then disseminated the participant information sheets to any members of the lung cancer clinical team who were eligible to participate. A participant information sheet was also distributed to lung cancer patients and carers who were eligible to take part. Participants had the option of either providing paper‐based consent and completing a demographics form at their participating NHS site or providing online consent and demographics information using a Google Form. Once consented, the central research team contacted the participants to arrange a suitable time and date for them to attend one of the online focus groups. Support with managing online participation in the focus groups was offered to participants. Recruitment was also promoted via national patient and/or nursing networks including Lung Cancer Nursing UK, the British Thoracic Oncology Group, The Roy Castle Lung Cancer Foundation, The Ruth Strauss Foundation, The Scottish Lung Cancer Nursing Forum and through a social media advert posted on *X*.

#### 4.1.4 Inclusion and/or Exclusion Criteria

For details of the inclusion and exclusion criteria for the focus groups, see Table 1.

**TABLE 1:** Eligibility criteria for focus group participants.

Inclusion criteria for healthcare professionals	Exclusion criteria for healthcare professionals
Actively involved in the clinical care pathways of lung cancer patients	Not actively involved in frontline clinical care
Working at one of the eight participating NHS hospitals	Not involved in caring for lung cancer patients for at least 30% of their role
Age between 18 and 75 years of age	
Inclusion Criteria for Patients and Carers	Exclusion Criteria for Patients and Carers
Must have a current diagnosis of lung cancer (any stage) or be the named carer of a person with a lung cancer diagnosis	Mesothelioma diagnosis or caring for someone with a mesothelioma diagnosis
Under the care of (or caring for) one of the participating NHS hospitals lung cancer teams	Unable to provide informed consent
Aged 18 or over	

#### 4.1.5 Data Collection

Seven focus groups were held, three for patients and carers, and four for healthcare professionals (*n* = 60). Eight participants took part in both the first and second patient and carer focus groups, and six took part in the third group. Six participants took part in the first focus group for healthcare professionals, five in the second, 13 in the third, and 14 in the final group. Eight potential participants consented but did not take part in a focus group because the date and time they were allocated were not convenient. Researchers were satisfied that data saturation had been reached and that it was not necessary to hold additional focus groups to accommodate these participants.

All focus groups were conducted online using the *Zoom* platform, with clinicians usually attending from their place of work, and patients and carers attending from their homes. At the start of the focus group, participants were reconsented verbally and were informed that the focus group would be recorded, using the recording function on *Zoom*. Each focus group lasted approximately 1 h and was facilitated and moderated by two members of the research team (CH, BL, ZD, IW, CD). The purpose of the overall study was explained to participants at the beginning of the sessions. Focus groups were not attended by anybody other than the participants and the researchers. Separate topic guides were developed for the healthcare professional and the patient/carer focus groups. Examples of questions contained within the topic guides include:

What do you think are some of the key challenges that can prevent nurses from speaking to lung cancer patients about clinical trial entry? And the solutions to these challenges?

What sorts of things would be useful to see in a resource that was aimed at promoting discussions between nurses and lung cancer patients about clinical trial opportunities?

#### 4.1.6 Data Analysis

Audio recorded focus group data were analysed thematically; this process was initiated by the data being transcribed by a transcription company with a local confidentiality agreement in place. All data were de‐identified at the point of transcription and any data from the focus groups was stored securely on a password protected Google Drive hosted by the participating university. Following deidentification, focus group data were analysed thematically by three coders: BL, CD, and ZD, using the Framework Method (Gale et al. 2013). Microsoft Excel was used as a data management tool. This method for systematically managing and analysing qualitative data allows researchers to compare and contrast data both within and across participant groups. The Framework Method consists of seven steps which were followed by the team. First, focus group data were transcribed by a transcription company with a local confidentiality agreement in place. Second, focus group recordings were listened to and read and reread; third, the transcripts were coded, and a selection double coded; fourth, a working analytical framework was developed by agreeing which codes should be applied to subsequent transcripts and by grouping together codes and categories. Fifth, the working analytical framework codes were applied to subsequent focus group transcripts to ensure consistency across the dataset. Sixth, two framework matrices were generated in Microsoft Excel, one for nurse participant focus groups and one for patient and carer focus groups. Coded data from the transcripts were then charted into the matrix, allowing data to be summarised concisely, without losing its meaning or richness. This inductive analysis process was complemented by deductive analysis techniques. Data were mapped into the matrices against the COM‐B model of behavioural change components (Michie et al. 2011) to provide a theorised understanding of the barriers and facilitators that impacted both healthcare professionals' and patient and carers' behaviours relating to clinical trial entry. Finally, the data were interpreted by the researchers through regular team discussions to generate themes and concepts relating to the research question.

#### 4.1.7 Ethical Considerations

National Health Service Research Ethics Committee (REC) and Helath Research Approval (HRA) permissions for the study “improving access and recruitment to clinical trials for lung cancer patients”, which covers the activities reported on in this paper, were obtained in May 2023, reference: REC: 23/EM/0094; IRAS ID: 325757. Oxford Brookes Univeristy approved sponsorship in March 2023, Ref: HLS.NHSS.23.2. Local Research and Development permissions were provided from nine participating NHS sites across England and Scotland. Of the nine sites, eight took part in the focus groups and co‐production workshop reported on in this paper.

Participants were not provided with compensation for taking part in the focus groups. Their privacy was protected through deidentification of the qualitative focus group datasets at the point of transcription. Participants were free to withdraw from the study at any time without giving a reason.

#### 4.1.8 Rigour and Reflexivity

The core research team included a Professor of Nursing and a Professor of Psychology, a Research Fellow and a Research Assistant, all of whom have PhDs, and a Research Assistant with an MSc and MA. Four were experienced qualitative researchers who had previously worked with cancer patients (CH, ZD, IW, BL). The fifth (CD) was a post‐doctoral researcher with developing expertise in mixed methods. The team contained two females and three males, and all had previous experience of conducting focus groups. The research team had minimal interaction with participants prior to the study commencing, and each introduced themselves, their credentials and experience, and interest in the topic area at the outset of the focus groups. To aid reflexivity, rigour, and trustworthiness of the research process, and to minimise any potential biases that might influence the analysis and interpretation of the findings, two researchers conducted the focus groups and debriefed following each group meeting. A selection of transcripts was double coded, and regular research team discussions aided interpretation of the study findings through the discussion of analytical approaches and the refinement of themes and concepts relating to the study findings.

### 4.2 Findings

#### 4.2.1 Characteristics of Participants

Sixty focus group participants were recruited in total, from across the eight NHS sites (*n* = 54), as well as through patient and nursing networks (*n* = 5) and social media (*n* = 1).^1^ Seventeen were lung cancer patients and five were carers of lung cancer patients. Thirty‐eight were healthcare professionals who worked with lung cancer patients. The size of the focus groups ranged from five participants to fourteen. See Tables 2a and 2b for participant characteristics.

**TABLE 2a:** Characteristics of healthcare professional participants (Particpants that consented but did not take part in focus groups are not included in the totals or the demographic tables).

Participant characteristics		*n* (%)
Age	18–29	1 (3)
	30–39	6 (16)
	40–49	12 (32)
	50–65	18 (47)
	66–75	1 (3)
Ethnicity	Any other ethnic group	1 (3)
	Asian or Asian British—Any other Asian background	1 (3)
	Asian or Asian British – Indian	3 (8)
	Asian or Asian British—Pakistani	1 (3)
	Black or Black British ‐ Caribbean	1 (3)
	Chinese	1 (3)
	White British	28 (74)
	White – Any other White background	2 (6)
Gender	Man	5 (13)
	Woman	33 (87)
Region	Midlands	9 (24)
	Northern England	2 (6)
	Northwest England	7 (18)
	Scotland	5 (13)
	Southern England	15 (39)
Professional Background	Doctor	6 (16)
	Lung Cancer Nurse	21 (55)
	Nurse Other	3 (8)
	Other	1 (3)
	Physio	1 (3)
	Research Nurse	6 (16)
Years working with cancer patients	1–5 years	7 (18)
	6–10 years	8 (21)
	More than 10 years	23 (61)
Total		38 (100)

**TABLE 2b:** Characteristics of patient and carer participants.

Participant characteristics		*n* (%)
Patient/Carer Role	Patient	17 (77)
	Carer	5 (23)
Age	18–34	0 (0)
	35–50	2 (9)
	51–65	6 (27)
	66–74	10 (45)
	75 and over	4 (18)
Ethnicity	Asian or Asian British – Bangladeshi	1 (5)
	Asian or Asian British—Indian	2 (9)
	White British	19 (86)
Gender	Man	6 (27)
	Woman	16 (73)
Region	Midlands	4 (18)
	Northern England	3 (14)
	Northwest England	9 (41)
	Southern England	5 (23)
	Scotland	1 (5)
Main hospital base	District General Hospital	6 (27)
	Teaching Hospital	16 (73)
Clinical trial opportunities discussed previously	Yes	7 (32)
	No	13 (59)
	Not sure	2 (9)
Previous participation in clinical trial	Yes	3 (14)
	No	17 (77)
	Not sure	2 (9)
Total		22 (100)

#### 4.2.2 Barriers and Facilitators to Lung Cancer Clinical Trial Enrolment

**FIGURE 2:**
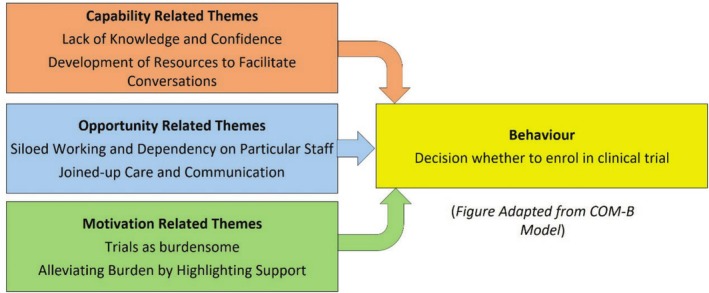
Six identified themes mapped using the COM‐B model.

Six themes were identified from the analysis process, using the COM‐B Framework (Michie et al. 2011) as a theoretical backdrop to aid understanding in relation to the initiation of clinical trial discussions between lung cancer nurses and patients (Figure 2). Each theme is described in more detail below.

#### 4.2.3 Capability Related Themes

##### 4.2.3.1 Theme 1 Lack of Knowledge and Confidence

Many healthcare professional participants voiced having low confidence and experiencing difficulties in explaining the concept of clinical trials to lung cancer patients, particularly when trying to explain different trial terminologies. Consequently, some reported a hesitancy in initiating discussions about trials with patients:

As a nurse, I didn't feel very confident sometimes talking about the lung trials […] I didn't feel like I had the knowledge to talk confidently (Healthcare Professional, Focus Group 2)

This hesitancy seemed to be reinforced by most lung cancer patients and carer participants, who reported little‐to‐no discussion of clinical trials with their cancer care teams at any time. This in turn left patients and carers stating that they had little understanding of trials or options to take part in them:

I've got no idea what trials there are with lung cancer […] I've got no understanding of trials. There's not been any discussion. (Lung Cancer Patient, Focus Group 5)

##### 4.2.3.2 Theme 2 Development of Resources to Facilitate Discussions

Many healthcare professional participants called for the development of a tool to help support their discussions with patients about clinical trial entry and to help structure and explain clinical trial concepts to patients:

I think they're [written tools] a really important adjunct to the verbal conversation that we have because patients would take away a very small portion of what you said to them because we forget stuff…So that is really the resource that you want them to read and reflect on and think about questions when you come back and have that next iterative conversation about the study (Healthcare Professional, Focus Group 3)

Similar views were shared by patients and carers, who commented that there was a need for more accessible and readily available clinical trial tools. Indeed, while many patients and carers had not themselves been signposted to trials, there was unanimous agreement between them that this should be done and that resources which succinctly explained the broad concepts of clinical trials would be advantageous to this:

‘Clinical trial’ and ‘research’ are quite probably alien words to most people […] So, I think just a really basic explanation of what research is and what clinical trials are just to help people understand how it might benefit […] them and…people in the future. (Lung Cancer Patient, Focus Group 5)

#### 4.2.4 Opportunity Related Themes

##### 4.2.4.1 Theme 3 Siloed Working and Dependency on Particular Staff

Many healthcare professional participants reported several organisational and resourcing issues that limited opportunities for them to discuss clinical trials with patients. These included issues related to staff numbers and workload, as well as ‘siloed’ work practices that resulted in little communication between research teams and care clinical teams. Together, these issues appeared to impede the prospect for healthcare professionals to present and discuss clinical trials with patients properly:

[The Research Nurse] did used to have a presence in our MDT, and obviously they made us aware of what trials were available, but that hasn't happened for some time now, so we're not always made aware. (Healthcare Professional, Focus Group 1)

A few patient and carer participants also commented on some of the pressures that healthcare professionals were under and how it could impact on their ability to discuss trials with patients:

The lung nurses are moving around a lot or they're only on part‐time or they're not given enough hours. And if they've got a lot [to do]…Will they have enough time […]? Because they seem to be under a lot of strain (Lung Cancer Patient, Focus Group 7)

##### 4.2.4.2 Theme 4 Joined‐Up Care and Communication

Responding to broader organisational and resourcing issues, healthcare professional participants spoke of the need for more coordinated approaches to care that included increased communications between research teams and clinical teams. Examples of joined‐up approaches to care were evidenced by some participants and were reflected on positively by those working at research centres of excellence:

The development of relationships between the clinical team and the research team is probably important. […] I've understood in my own situation I need to make a better attempt at that as well. And I'm a Senior Lung Cancer Nurse. And it's how do we make that engagement with the research teams to […] come and speak to the nurses (Healthcare Professional, Focus Group 2)

The need for a more integrated and collective approach to care, to help distribute responsibility, and increase opportunities for healthcare professionals to discuss trials was similarly discussed by patient and carer participants:

It should be more of a collective thing where… the lung nurse team and […] various people that are in that whole team… make the whole thing work, really. It's not really down to one person when you think about it. It is a team issue, isn't it? I mean, with the doctors and the nurses together. (Lung Cancer Patient, Focus Group 4)

#### 4.2.5 Motivation Related Themes

##### 4.2.5.1 Theme 5 Trials as Burdensome

Several healthcare professional participants reported that they thought that some patients were put‐off by the perception of trials as being more burdensome than the current standard of care, and that they might be less likely to take part due to the added commitments or perceived cost implications that were often involved:

It's the extra visits, the potential just new trials, are more onerous compared to standard of care, and that can put people off, especially when they've got caring responsibilities either for children or their partner who might be sick (Healthcare Professional, Focus Group 3)

This perspective was echoed by several patient and carer participants:

I think a lot of people may […] find it impossible to participate in some of these clinical trials because of logistical, personal, financial considerations (Lung Cancer Patient, Focus Group 5)

##### 4.2.5.2 Theme 6 Alleviating Burden by Highlighting Support

Most healthcare professional participants recognised that patients and carers might be deterred from taking part in a trial due to the associated additional travel and visits. They stressed the need to highlight to patients any available support that was on offer:

We used to have hotels that would give reduced rates for patients. So, even if they had to pay, it was a reduced rate, and it was quite close to where they needed to attend. So, it's looking at all of that as well, it's the bigger picture, not just, this is a trial, let's get you recruited. This is what we can offer you to help you to get there. (Healthcare Professional, Focus Group 6)

This perspective was also shared and voiced by several lung cancer patient and carer participants:

Trying to highlight that there's certain aspects that would encourage people to come along. Like transportation, payments, helping to get to the hospitals (Lung Cancer Patient, Focus Group 7)

## 5 Phase 2: Tool Development

### 5.1 Methods

#### 5.1.1 Summary of Tool Development Process

Development of the intervention tool was underpinned by rigorous processes, in alignment with the Medical Research Council's Framework for the Development of Complex Interventions (Skivington et al. 2021; O'Cathain et al. 2019). This involved consulting the relevant international literature and undertaking primary research, using focus groups, to inform initial intervention development. Consultation with stakeholders, including nurses, wider multi‐disciplinary team members, patients, and carers, provided useful feedback on the intervention prototype. Further intervention development and refinement were then undertaken through this iterative consultation process until consensus was reached on the final Lung I‐ACT intervention.

#### 5.1.2 Logic Modelling and Theoretical Framework

Findings from both the systematic review (Lond et al. 2024) and the focus groups were used to aid the development of a prototype Lung I‐ACT tool. The researchers created a logic model (Smith et al. 2020) to develop practical mechanisms for influencing behaviour change as identified through the COM‐B analysis exercise (Michie et al. 2011). The COM‐B theory of behaviour change, along with the Behaviour Change Wheel, was utilised to develop the findings which underpinned the content of the tool (Michie et al. 2011). The Wheel allowed the researchers to identify interventions that could help facilitate a behaviour change of both lung cancer patients and lung cancer nurses in relation to discussing and considering clinical trial enrolment.

#### 5.1.3 Co‐Production and Stakeholder Involvement

The Lung I‐ACT prototype tool consisted of both patient‐facing and nurse‐facing elements. These were further developed using a co‐production method involving input from study co‐applicants and collaborators during online meetings (Martin 2010; Henshall et al. 2018; Smith et al. 2022). Various iterations of the Lung I‐ACT tool were reviewed for content and face validity by these stakeholders, who included academics, lung cancer nurses, and a lung cancer patient advocate. Suggested modifications were incorporated into the tool. These modifications included using the first person more frequently to promote engagement and ensuring that the terminology and language were pitched at the right level and covered the right topic areas.

The updated Lung I‐ACT prototype was then shared with both lung cancer patients (*n* = 2) and lung cancer nurses (*n* = 7) during a co‐production workshop which lasted 3 h and took place online via the *Zoom* platform. The workshop was recorded so that researchers could ensure the suggestions were accurately incorporated. The discussion focused on four key areas: content, layout, format, and level of detail required. Workshop members also commented on ways that the elements of the tool could be best implemented within the clinical setting.

#### 5.1.4 Tool Refinement

The final iterations of the tool were produced in collaboration with the core research team, the wider co‐applicant and collaborator group, and a graphic designer, actioning the feedback garnered from the workshop and mapping these against the outcomes of the COM‐B behaviour change wheel and logic model exercises (Tables 3a and 3b). This helped to further refine the Lung I‐ACT tool ready for piloting at the participating NHS sites.

**TABLE 3a:** A summary of key aspects of the logic model that informed the content and design of the patient‐facing element of the tool using the COM‐B Model of Behavioural Change.

Identified problem	Suggested feature/modification to address problem	Outcomes as mapped against COM‐B model
Patients do not know what a clinical trial is or what it involves. General public perceive trials as risky/strenuous	Basic description of clinical trial purpose and process Diagram to illustrate clinical trial enrolment process Answer FAQs	Increase Opportunity for discussion about clinical trials Enhance Capability of nurses and patients to discuss clinical trials
Some patients have below average reading age	Short sentences/simple vocabulary	Increase Capability of patients to learn about clinical trials
Patients unaware of benefits of clinical trial enrolment Patients likely to enrol because of altruistic/hope motivations	List altruistic/clinical/interpersonal motivations for taking part in trial	Provide Motivation for patients to seek information about clinical trials
Patients do not understand clinical research terminology	Use simple, “lay” terminology where possible	Enhance Capability of patients to learn about clinical trials
Cancer nurses concerned they might be unable to explain research terminology.	Glossary of frequently used terms	Increase Opportunity for patients to learn about clinical trials
Clinical lab‐based images are off‐putting Images of a “friendly face” and/or nurse are reassuring	No images of medical or scientific equipment Use illustrations rather than photographs Ensure illustration is of a nurse	By making tool more accessible Capability of patients to learn more about clinical trials increases
Clinical trial conversations take up valuable time	Produce pamphlet that is easy to use in practice	Increase Opportunity for nurses to introduce the concept of trial to patients
Not all patients/staff are digital natives	Produce as paper copy, with digital option	Enhance Capability of nurses and patients to access information abour clinical trials
Patients can be overwhelmed with information	Designed for patients to take home and read	Increase Opportunity for patients to learn about clinical trials
Patients unaware of where to find additional trial information Patients unaware of trial opportunities available	Include links to webpages with more detailed information about trials	Increase Capability of patients to access trial information
Blocks of text difficult to focus on Bullet points easy to read	Reduce block test and increase use of visuals. Optimise level of detail. Bullet points where appropriate	Increasing usability of tool increases Capability of patients to access trial information

**TABLE 3b:** A summary of key aspects of the logic model that informed the content and design of the nurse‐facing element of the tool using the COM‐B model of behavioural change.

Identified problem	Suggested feature/modification to address problem	Outcomes as mapped against COM‐B model
Nurses can lack knowledge of clinical trials	Prompt nurses to use patient‐facing element for relevant information Reassure nurses that aim is to signpost patients; detail not required	Enhance Capability of nurses to have a conversation about clinical trials by providing and signposting information Increase Opportunity for nurses to discuss clinical trials by prompting them Provide Motivation for nurses to have conversation about clinical trials by reassuring them they do not need to have detailed knowledge
Nurses do not view it as part of their role to discuss clinical trials	Prompt to encourage conversations and their benefits	Provide Motivation for nurses to discuss clinical trials through encouragement
Limited communication between clinical and research teams	Promote regular communication methods	Increase Capability of nurses to discuss clinical trials through encouraging cross‐departmental communication
Nurses unsure of correct time to discuss clinical trials with patients	Clarify that good to discuss at all stages of patient pathway Encourage “drip‐feed” approach Use patient‐facing element to initiate conversations	Provide Opportunity for nurses to discuss clinical trials through encouragement
Tool should be simple and to the point Should act as a “prompt”	No long sentences or detail. Key messaging accentuated	Provide Opportunity for nurses to discuss trials by targeted prompts
Time pressures make too much information difficult to absorb Tool needs to be simple	Wall poster best way to convey information	Increase Opportunity to have discussions about clinical trials by being simple and targeted
Limited geographic availability of trials	Wipe‐clean section for adding local trials information	Increase Capability of nurses to have discussions by providing them with information on trial availability
Nurses lack time to read lots of information	Simple messaging to encourage use of patient‐facing element	Increase Opportunity for nurses to have conversations about clinical trials by making it information accessible to them and to patients

### 5.2 Findings

Data from a previously published systematic review (Lond et al. 2024), focus groups and co‐production stakeholder workshops uncovered many findings that fed into the development of the Lung I‐ACT tool. Firstly, the data confirmed that most patients had minimal knowledge of what clinical trial participation involved and had not had any discussions about clinical trials with members of their healthcare team (Health and Social Care Committee 2022). Secondly, a lack of self‐efficacy amongst lung cancer nurses in discussing clinical trial opportunities was identified. This was underpinned by the perception that they lacked the necessary knowledge to initiate such discussions, something that is supported in the wider literature (McPhelim 2015). These findings led to the Lung I‐ACT patient‐facing element of the tool being designed to provide basic trial information and a structure for discussions between lung cancer nurses and patients. The prototype of this element was designed as a pamphlet; this was confirmed as the preferred format during the co‐production workshop. It was also agreed that, whilst it could be available digitally, it should be fully functional as a paper‐based pamphlet to ensure usability for both nurses, patients, and carers (see Table 3a). It also informed the decision to create the second, nurse‐facing element of the Lung I‐ACT tool. This was initially designed as an information sheet but was adapted following consultation in the co‐production workshop to be a large poster that could be easily displayed in a prominent position in nurses' staff rooms, offices or clinical areas (see Table 3b). The purpose of this element was to remind nurses that they did not need detailed knowledge of clinical trials to initiate discussions, and that they could use the patient‐facing element as a tool to answer patients' questions. It also aimed to encourage communication between lung cancer nursing teams and research delivery nursing teams, as a means of promoting confidence and knowledge in talking about local trial opportunities with patients and the wider healthcare team.

The features of the prototype design and subsequent modifications to both the nurse‐ and patient‐facing elements of the Lung I‐ACT tool are detailed in Tables 3a and 3b and present an overview of the logic model exercise taken from each stage of the project. The tables also explain the intended outcomes of the tool's features mapped on to the COM‐B model of behavioural change that underpinned the decisions made.

## 6 Discussion

The Lung I‐ACT tool is the first co‐produced, complex intervention developed specifically for use in the clinical setting to support the discussion of clinical trial opportunities between lung cancer nurses and patients and makes an original contribution to the field. This study adds to the evidence base by supporting the contention that the development of person‐centred tools that promote discussions about clinical trials is supported by cancer nurses, other multidisciplinary team members, cancer patients and their carers (Unger et al. 2019; The Institute of Cancer Research 2021). By identifying barriers to discussions about trials and supporting nurses to have the knowledge, confidence, and awareness to overcome them, the Lung I‐ACT tool can facilitate conversations to help patients make informed choices about clinical trial enrolment. A recent national cancer patient experience survey revealed that almost half of lung cancer patients reported that they did not recall having discussions about research participation with a healthcare professional (NHS England 2023). The Lung I‐ACT tool aims to address this by increasing the quality and quantity of research discussions between nurses and patients; this can ultimately lead to increased trial participant recruitment numbers.

Use of the COM‐B theoretical framework of behavioural change (Michie et al. 2011) during the research process enabled the identification of different ways that nurses' behaviours might be influenced in terms of the quantity and quality of discussions they have with lung cancer patients about clinical trials. Factors influencing nurses' capabilities, opportunities, and motivations to have conversations about clinical trials fed directly into the design of the tool to make it as effective, theoretically informed, and grounded in the evidence base as possible.

Focus groups with nurses identified that a lack of knowledge and confidence underpinned nurses' reduced capabilities to discuss trials with patients. Therefore, the Lung I‐ACT tool serves to remind and reassure lung cancer nurses that they are not expected to be experts in clinical trials or to hold the most up‐to‐date information about which trials are available to their patients. Rather, the tool, which contains simple language and useful visual aids, can act as a prompt and signposting mechanism by which to initiate conversations with patients about clinical trials that might otherwise be omitted, increasing the likelihood of meaningful conversations taking place (Michie et al. 2011; Collet et al. 2022). The Lung I‐ACT tool has been designed by acknowledging enabling and hindering factors that influence patient decision‐making around clinical trial entry; these have been identified in the literature and through the focus group findings described in this paper (Dodd et al. 2024; Lond et al. 2024). As such, the tool can address and overcome some of these issues. It promotes meaningful engagement in discussions on the topic of clinical trials, supporting nurses by addressing any barriers they have identified, whilst incorporating information that patients have cited as informing their own behaviours related to clinical trial enrolment.

The findings that have been generated are person‐centred, with the potential to make significant improvements to patient decision‐making around trial entry in future. Co‐production was employed throughout the process to ensure the tool was designed to meet the needs of both lung cancer nurses and lung cancer patients and, crucially, that they were practical for use in a clinical setting (Henshall and Davey 2020; Smith et al. 2022). This is likely to increase the future acceptability and feasibility of implementing the tool in busy cancer care settings. Co‐production is known to benefit intervention design as the expertise of stakeholders can be utilised to inform the decision‐making process (Henshall et al. 2018; Smith et al. 2022). In this case, strong stakeholder involvement from the conception of the study through to the final tool being developed ensured the tool was feasible and acceptable to both lung cancer nurses and lung cancer patients. Specifically, input from both patients and nurses led to the identification of the need for two complementary nurse‐and patient‐facing elements to complete the Lung I‐ACT tool. The patient‐facing element has been designed as a three‐fold pamphlet, based on the findings that the tool would need to be convenient for nurses to carry and for patients to take home. This form of written information is further enhanced if supported by a verbal explanation from nurses as the use of both synchronous and asynchronous communication is known to be effective in communicating treatment options to patients (Kang et al. 2020). The preference for the nurse‐facing element was for it to take the form of a poster that would act as a prompt and quick, easy‐to‐use reference point for more information. Co‐production with stakeholders also identified a preference for both elements of the tool to be designed as hard copy resources rather than digital‐only. It was felt by both nurses and patients that digital‐only resources would exclude some individuals and reduce the potential efficacy of the tool. The two complementary elements have been designed for use in conjunction with one another, with the aim of maximising their reach, usefulness, and versatility, rather than reverting to a one‐size‐fits‐all approach that lessens the utility of the tool for nurses and lung cancer patients alike. This has been shown to be effective in other educational and informational tools within healthcare settings (Bomhof‐Roordink et al. 2019).

Whilst the Lung I‐ACT tool has been designed for use by lung cancer nurses, lung cancer patients, and their carers, the versatility of the tool and the diversity of stakeholders that have contributed to its design means that it has the potential to be adopted for use by other members of the multidisciplinary team. Both elements of the tool can be displayed in staff areas, office spaces, and in a variety of clinical areas, and the information contained within them can be utilised by all members of the cancer care team, regardless of their professional role, setting, or level of experience. The tool also has the potential to be transferable to other healthcare settings, such as other cancer sites or respiratory care, provided relevant, disease‐specific changes to the tool are made to account for contextual, setting‐specific factors that differentiate lung cancer from other specialties. It also has the potential to be adopted outside of the UK and to inspire similar interventions in healthcare systems internationally.

### 6.1 Strengths and Limitations

This study was a national, multisite study that recruited stakeholders from a range of teaching hospitals and District General Hospitals across England and Scotland. As such, the nurses, multidisciplinary team members, patients and carers who participated in the study had experiences of working and being cared for in settings with different geographical locations, socio‐demographic backgrounds, hospital sizes and settings, and with differing levels of clinical trials availability. Some of the sites involved had little experience of running lung cancer clinical trials; whereas other sites were recruiting lung cancer patients to trials on a daily basis. This variation has informed the development of the Lung I‐ACT tool, increasing its versatility and transferability across a range of different contexts and settings.

The process of developing the Lung I‐ACT tool has been shaped by current models of good practice in health intervention design (Skivington et al. 2021; O'Cathain et al. 2019). We have collected a significant corpus of primary data from a relatively large, diverse sample of patients and professionals, and synthesised this with a systematic review of the extant literature to identify the core challenges in recruiting lung cancer patients to clinical trials. The robust co‐production element threaded throughout this study serves to enhance the person‐centred nature of the work and subsequent tool that has been developed (Henshall et al. 2018). Patient advocates are included as co‐authors on this paper, adding to the rigour and transparency of our approach and the co‐production process. Furthermore, the Lung I‐ACT tool and its logic model is underpinned by the COM‐B theoretical behaviour change framework, enhancing its validity and trustworthiness and increasing the credibility of the complex intervention in the longer term.

Our focus group patient sample was geographically dispersed and included patients and staff from centres with above and below average rates of lung cancer clinical trial participation. We were able to over‐recruit to focus groups, enabling us to reach data saturation; this aided our confidence in the reliability and trustworthiness of the findings. Although clinical trial literacy across the focus groups was mixed, participants were likely to have been interested in learning about clinical trial opportunities. In terms of focus group demographics, more patient and carer participants were women (*n* = 16) than men (*n* = 6) despite men having marginally higher lung cancer incidence rates (52%) than women (48%) in the UK (Cancer Research UK 2025). In addition, recruitment from non‐White ethnocultural groups was not as broad as it could have been. Whilst there is a lower prevalence of lung cancer amongst non‐White populations (Delon et al. 2022), it is important to gather data from across all ethnocultural groups to ensure their views are represented in the research process. This is particularly important given that Black individuals tend to have lower rates of participation in clinical trials than other ethnocultural communities (Grant et al. 2020). This may be for several reasons, including experiences of systemic racism in healthcare and an associated scepticism about taking part in healthcare innovations (Hamed et al. 2022).

### 6.2 Implications for Policy and Practice

The Lung I‐ACT tool is a versatile, interactive nurse and patient focused resource for nurses to use in their daily practice alongside patients. It has been designed to be incorporated into everyday clinical practice, being time‐efficient and easy to incorporate into discussions with lung cancer patients and their carers. The tool has been designed for nurses and patients *by* nurses and patients and has been created in different formats to increase accessibility and reach. Nurses have reported feeling underconfident when talking to patients about clinical trial entry (McPhelim 2015); the Lung I‐ACT tool can be a simple yet effective way of removing any issues relating to confidence. Instead, use of the tool can empower nurses to have conversations about trials with patients so that they can make informed choices about their treatment and care pathways (NICE 2024).

Clinical trial recruitment figures across the UK and globally have fallen over the past decade, especially in cancer care settings (Health and Social Care Committee 2022). The Lung I‐ACT tool is one way to help improve the recruitment of lung cancer patients into clinical trials. The tool aims to facilitate better conversations about trial entry between nurses and patients, as well as promote more joined‐up communication pathways between lung cancer nurses and other members of the lung cancer research team, such as research nurses and principal investigators. This promotion of enhanced communication pathways can facilitate the production of more widespread knowledge and awareness of clinical trials across lung cancer nursing teams as well as promote more holistic, patient‐centred care pathways (Kruijver et al. 2000; Collet et al. 2022).

### 6.3 Recommendations for Further Research

Future research will focus on examining the acceptability of implementing the Lung I‐ACT tool across clinical settings. The study team will conduct a multi‐site pilot study across England and Scotland to test the acceptability of the Lung I‐ACT tool and identify whether it can enhance lung cancer nurses' knowledge, confidence, and awareness about having discussions with lung cancer patients about clinical trial entry. The pilot study will also elicit the views of patients who have been offered clinical trials to identify if they were satisfied with the discussions that took place. If the pilot study findings show that the Lung I‐ACT tool is acceptable for use, it has the potential to be adopted for widespread use across healthcare settings.

## 7 Conclusion

The Lung I‐ACT tool that has been generated from the processes reported on in this paper has the potential to make a significant impact on recruitment to cancer clinical trials across a wide range of clinical care settings and sites. The person‐centred tool has been rigorously designed using co‐production methods and has widespread, global application potential.

By identifying barriers and facilitators to lung cancer clinical trial enrolment and utilising these findings to inform the development of a person‐centred intervention that has practical applications in the cancer care setting, the Lung I‐ACT tool can increase and improve the discussions that lung cancer nurses have with patients about clinical trial opportunities. This could lead to more lung cancer patients choosing to take part in clinical trials, with potentially promising outcomes for both patients themselves, trial recruitment figures, and the wider UK life sciences industry (Health and Social Care Committee 2022; Dodd et al. 2024). The tool promotes informed decision making for lung cancer patients as well as improving nurses' knowledge, confidence, and awareness about discussing clinical trials with patients. Future research will pilot the use of the Lung I‐ACT tool across the UK to determine its acceptability for use in clinical care settings.

## Author Contributions

C.H., C.D., B.L., Z.D., I.R.W., L.D., S.H., J.M., J.R.: Made substantial contributions to conception and design, or acquisition of data, or analysis and interpretation of data; C.H., C.D., B.L., Z.D., I.R.W., L.D., S.H., J.M., J.R.: Involved in drafting the manuscript or revising it critically for important intellectual content; C.H., C.D., B.L., Z.D., I.R.W., L.D., S.H., J.M., J.R.: Given final approval of the version to be published. Each author should have participated sufficiently in the work to take public responsibility for appropriate portions of the content. C.H., C.D., B.L., Z.D., I.R.W., L.D., S.H., J.M., J.R.: Agreed to be accountable for all aspects of the work in ensuring that questions related to the accuracy or integrity of any part of the work are appropriately investigated and resolved.

## Conflicts of Interest

The authors declare no conflicts of interest.

## Supporting information


**Data S1:** jan70134‐sup‐0001‐Supinfo.docx.

## Data Availability

The data that support the findings of this study are available from the corresponding author upon reasonable request.

## Endnotes

^1^
Particpants that consented but did not take part in focus groups are not included in the totals or the demographic tables.
