# Supplementary material for: Improving Access and Recruitment to Clinical Trials for Lung Cancer Patients: A Multi‐Phase, Qualitative Focus Group and Co‐Production Study
Source: J Adv Nurs. 2025 Aug 14;82(5):5096–109. doi: 10.1111/jan.70134 (PMC13069228; doi:10.1111/jan.70134)
Supplement: Supplementary file 1 — Data S1: jan70134‐sup‐0001‐Supinfo.docx. [file JAN-82-5096-s001.docx]

# Consolidated criteria for reporting qualitative studies (COREQ): 32-item checklist

Adapted from Tong, A., Saisnbury, P., Carig, J. (2007) Consolidated criteria for reporting qualitative research (COREQ): a 32-itemchecklist for interviews and focus groups. *International Journal for Quality in Health Care*; 19, 6, 349–357.

| **No.** | **Item** | **Description** | **Page reference** |
| --- | --- | --- | --- |
| **Research team and reflexivity** | | | |
| Personal characteristics | | | |
| *1.* | Interviewer/facilitator | Which author/s conducted the interview or focus group? | page 8 |
| *2.* | Credentials | What were the researcher's credentials? *E.g. PhD, MD* | page 10 |
| *3.* | Occupation | What was their occupation at the time of the study? | page 10 |
| *4.* | Gender | Was the researcher male or female? | page 10 |
| *5.* | Experience and training | What experience or training did the researcher have? | page 10 |
| Relationship with participants | | | |
| *6.* | Relationship established | Was a relationship established prior to study commencement? | page 10 |
| *7.* | Participant knowledge of the interviewer | What did the participants know about the researcher? *E.g. Personal goals, reasons for doing the research* | page 10 |
| *8.* | Interviewer characteristics | What characteristics were reported about the interviewer/facilitator? *E.g. Bias, assumptions, reasons and interests in the research topic* | page 10 |
| **Study design** | | | |
| Theoretical framework | | | |
| *9.* | Methodological orientation and theory | What methodological orientation was stated to underpin the study? *E.g. grounded theory, discourse analysis, ethnography, phenomenology, content analysis* | pages 8-9 |
| Participant selection | | | |
| *10.* | Sampling | How were participants selected? *E.g. purposive, convenience, consecutive, snowball* | pages 7-8 |
| *11.* | Method of approach | How were participants approached? *E.g. face-to-face, telephone, mail, email* | page 7 |

| *12.* | Sample size | | How many participants were in the study? | | pages 9-10 | |  |  |
| --- | --- | --- | --- | --- | --- | --- | --- | --- |
| *13.* | Non-participation | | How many people refused to participate or dropped out? What were the reasons for this? | | pages 8 | |  |  |
| Setting | | | | | | |  |  |
| *14.* | Setting of data collection | | Where was the data collected? *E.g. home, clinic, workplace* | | page 8 | |  |  |
| *15.* | Presence of non-participants | | Was anyone else present besides the participants and researchers? | | Page 8 | |  |  |
| *16.* | Description of sample | | What are the important characteristics of the sample? E.g. demographic data, date | | page 11-12 | |  |  |
| Data collection | | | | | | |  |  |
| *17.* | Interview guide | | Were questions, prompts, guides provided by the authors? Was it pilot tested? | | page 9 and as a supplemental | |  |  |
| *18.* | Repeat interviews | | Were repeat interviews carried out? If yes, how many? | | n/a | |  |  |
| *19.* | Audio/visual recording | | Did the research use audio or visual recording to collect the data? | | page 8 | |  |  |
| *20.* | Field notes | | Were field notes made during and/or after the interview or focus group? | | no | |  |  |
| *21.* | Duration | | What was the duration of the interviews or focus group? | | page 8 | |  |  |
| *22.* | Data saturation | | Was data saturation discussed? | | page 8 | |  |  |
| *23.* | Transcripts returned | | Were transcripts returned to participants for comment and/or correction? | | No | |  |  |
| **Analysis and findings** | | | | | | |  |  |
| Data analysis | | | | | | |  |  |
| *24.* | Number of data coders | | How many data coders coded the data? | | pages 9 | |  |  |
| *25.* | Description of the coding tree | | Did authors provide a description of the coding tree? | | pages 9 | |  |  |
| *26.* | Derivation of themes | | Were themes identified in advance or derived from the data? | | pages 9 | |  |  |
| *27.* | Software | | What software, if applicable, was used to manage the data? | | pages 9 | |  |  |
| *28.* | | | Participant checking | | Did participants provide feedback on the findings? | | no | |
| Reporting | | | | | | | | |
| *29.* | | | Quotations presented | | Were participant quotations presented to illustrate the themes / findings? Was each quotation identified? *E.g. Participant number* | | Pages 13-16 | |
| *30.* | | | Data and findings consistent | | Was there consistency between the data presented and the findings? | | pages 13-16 | |
| *31.* | | | Clarity of major themes | | Were major themes clearly presented in the findings? | | pages 13-16 | |
| *32.* | | | Clarity of minor themes | | Is there a description of diverse | | pages 13-16 | |
